# Cryogenic Carbon Monoxide Oxidation on Cuprous Oxide

**DOI:** 10.1002/anie.202515673

**Published:** 2025-11-10

**Authors:** Burcu Karagoz, Tianhao Hu, Joakim Halldin Stenlid, Xiaoming Hu, Markus Soldemo, Frank Abild‐Pedersen, Kess Marks, Henrik Öström, Dario Stacchiola, Jonas Weissenrieder, Ashley R. Head

**Affiliations:** ^1^ Diamond Light Source Diamond House Didcot OX11 0DE UK; ^2^ Department of Chemistry Stony Brook University Stony Brook NY 11794 USA; ^3^ SUNCAT Center for Interface Science and Catalysis SLAC National Accelerator Laboratory Menlo Park CA 94025 USA; ^4^ SUNCAT Center for Interface Science and Catalysis Department of Chemical Engineering Stanford University Stanford CA 94305 USA; ^5^ Department of Physics AlbaNova University Center Stockholm University Stockholm SE‐106 91 Sweden; ^6^ Light and Matter Physics Applied Physics KTH Royal Institute of Technology Stockholm SE‐100 44 Sweden; ^7^ Department of Chemistry and Chemical Engineering Chalmers University of Technology, Gothenburg, SE‐412 96 Sweden; ^8^ Center for Functional Nanomaterials Brookhaven National Laboratory Upton NY 11973 USA

**Keywords:** CO oxidation, Cuprous oxide, Density functional calculations, IRRAS, Surface chemistry

## Abstract

Performing oxidation reactions at low temperatures using earth‐abundant materials is crucial for advancing solutions for sustainable chemistry. CO oxidation serves as a benchmark reaction to characterize oxidation and to advance fundamental concepts in surface chemistry. While there are several examples of CO oxidation occurring on metal oxides at low temperatures, from 300 K to ∼200 K, reactivity in the cryogenic temperature regime typically requires a metal nanoparticle on a metal oxide. Here, we show oxygen atoms on the (111) facet of Cu_2_O react with CO to form CO_2_ at temperatures below 100 K. Combining spectroscopic experimental evidence with calculations, we propose a low barrier path for CO oxidation at reconstructed surface sites on Cu_2_O(111). This finding is a rare example of an earth‐abundant metal oxide, in this case copper, that can provide highly reactive multifunctional sites, enabling both adsorption and reaction fundamental steps toward the efficient heterogeneous oxidation of chemicals.

## Graphical abstract

A reconstructed Cu_2_O surface with pyramidal units (PY) contains both adsorption and reaction sites for CO oxidation. A suite of spectroscopy techniques and calculations were used to characterize the fundamental surface chemistry occurring below 100 K, where typically a metal/oxide interface is required. This earth‐abundant metal oxide shows that local surface ensembles of atoms can vary in reactivity.

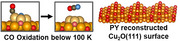

## Introduction

Finding efficient chemical reaction pathways that make use of earth‐abundant metals like iron or copper is essential for a sustainable future.^[^ ^1^ ^]^ A prototypical reaction used to explore the surface reactivity of materials toward oxidation reactions is the heterogeneous oxidation of CO, a benchmark reaction for fundamental studies and practical applications.^[^ ^2^ ^]^ Mechanistic studies of surfaces are often explored by measuring how CO interacts with a surface at low temperatures. Haruta and coworkers brought the low‐temperature oxidation of CO to the fore when they found that gold nanoparticles on metal oxides are efficient catalysts below room temperature.^[^ ^3^ ^]^ In some cases, metal monomers or dimers are needed to extract oxygen from the oxide substrate.^[^ ^4^ ^]^ Metal oxides themselves have shown reactivity for CO oxidation at low temperatures. While Co_3_O_4_ is one of the most active,^[^ ^5^ ^]^ mixed metal cobalt oxides, iron and mixed metal iron oxides,^[^ ^6^ ^]^ and MnO_2_^[^ ^7^ ^]^ also facilitate CO oxidation through a lattice oxygen pathway consistent with a Mars‐van Krevelen mechanism. In most of these cases, the low‐temperature regime spans from ambient to approximately 200 K. Very recently, the rare‐earth oxide ceria has been reported to oxidize CO below 100 K with a surface peroxide being a key intermediate.^[^ ^8^ ^]^

Earth‐abundant copper‐based materials are active toward the conversion of small molecules^[^ ^9^, ^10^ ^]^ with the oxidation state of copper sites and the local surface atom arrangement dictating the reaction selectivity. For example, the Cu_2_O(111) and Cu_2_O(100) surfaces have been shown to have dramatically different interactions with water and oxygen.^[^ ^11^, ^12^, ^13^, ^14^, ^15^, ^16^ ^]^ For the case of the Cu_2_O(111) surface, Gloystein et al. recently resolved a longstanding debate regarding the atomic structure for its (√3 × √3)*R30*° surface reconstruction, revealing a surface structure comprised of repeating nanopyramid units, denoted the PY reconstructed surface.^[^ ^11^ ^]^ While both CO and CO_2_ interactions have been studied experimentally and computationally, the studies largely considered the unreconstructed surface or reconstruction patterns associated with Cu_2_O thin films on supports.^[^ ^17^, ^18^, ^19^, ^20^, ^21^, ^22^ ^]^

Here, we present combined experimental and computational results of CO reacting with highly active O atoms on the PY reconstructed Cu_2_O(111) surface at cryogenic temperatures. The results are a rare example of reactivity of oxygen at temperatures below 100 K. This study demonstrates that an oxide, in this case from an earth‐abundant metal, can provide both sites for CO adsorption and oxygen atoms for its oxidation at exceptionally low temperatures.

## Results and Discussion

### CO_2_ Formation from CO and Surface Oxygen Atoms

**Figure 1:**
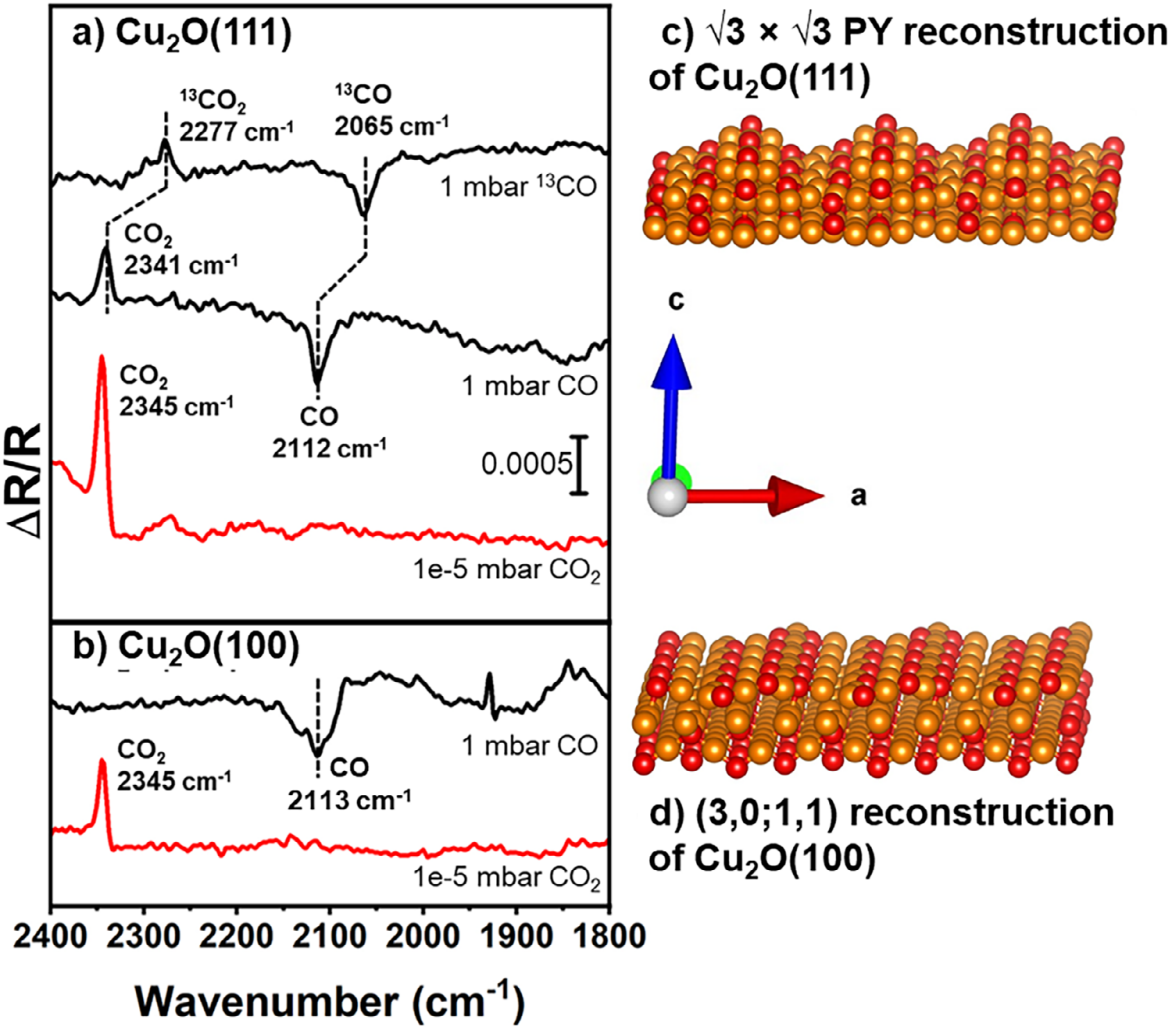
IRRAS data of the surface species a) on Cu_2_O(111) at the listed pressures of ^13^CO, CO, and CO_2_ and b) on Cu_2_O(100). All the spectra were collected below 140 K, with CO_2_ formation observed at temperatures as low as 100 K. c) PY reconstruction of the Cu_2_O(111) surface and d) the (3,0;1,1) reconstruction of the Cu_2_O(100) surface, with O atoms red and Cu atoms orange.

Vibrational spectroscopy allows distinguishing among different surface adsorbates and provides information on their specific adsorption sites and configuration. Infrared reflection absorbance spectroscopy (IRRAS) can be performed on surfaces under elevated pressures by using polarized light to remove gas phase contributions.^[^ ^23^ ^]^ Figure 1a plots the IRRAS data as a change in the Cu_2_O(111) surface reflectivity at cryogenic temperatures under a pressure of 1 mbar of CO compared to the reflectivity of the clean substrate in ultra‐high vacuum (Δ*R*/*R*). At this elevated CO pressure, the coverage of adsorbed molecules is maximized. A peak at 2112 cm^−1^ with negative intensity can be assigned to CO adsorbed on Cu^1+^ sites.^[^ ^24^, ^25^, ^26^ ^]^ This frequency is on the low end of the range of Cu^1+^–CO adducts reported,^[^ ^24^ ^]^ suggesting the most favorable adsorption site for CO on the reconstructed Cu_2_O(111) surface is slightly reduced. Surprisingly, CO reacts at these low temperatures with the surface, leading to an additional sharper feature at 2341 cm^−1^ with positive intensity, consistent with CO_2_ bound to a metal oxide surface.^[^ ^27^ ^]^ A similar feature is observed when Cu_2_O(111) is directly exposed to CO_2_ (red spectrum in Figure 1a). When CO_2_ is formed from CO, the frequency is 4 cm^−1^ lower than when CO_2_ is condensed on the surface, indicating that the reaction leads to a slightly modified surrounding that results in a stronger surface interaction with the produced CO_2_.

To confirm that CO_2_ is being formed by the reaction of adsorbed CO with oxygen from the surface, experiments with isotopically labeled ^13^CO were performed. A resulting 64 cm^−1^ shift for the observed CO_2_ IR feature (Figure 1a) matches the expected isotope‐induced shift.^[^ ^24^ ^]^ The CO peak assignment is also verified by the ^13^CO experiments, with a corresponding shift by 47 cm^−1^.^[^ ^24^ ^]^ The frequency of the adsorbed CO_2_ generated by CO oxidation indicates a weakly physisorbed molecule. No evidence is observed by the IR experiments for the presence of activated CO_2_, which is characterized by a feature at ∼1280 cm^−1^ that is typically less intense than the feature associated with molecular CO_2_.^[^ ^28^ ^]^

A Cu_2_O(100) surface was exposed to the same CO adsorption conditions to explore if CO_2_ formation on cuprous oxide surfaces is structure sensitive. This surface lacks the PY motif; instead, it features a (3,0;1,1) reconstruction with protruding rows aligned along the [011] direction and Cu trimer sites situated between these rows,^[^ ^14^, ^15^ ^]^ as shown in Figure 1d. The IRRAS data in Figure 1b show that CO adsorbs but does not form CO_2_. The red spectrum in Figure 1b shows that CO_2_ does adsorb on the Cu_2_O(100) from the gas phase at cryogenic temperatures, indicating that if CO_2_ were formed from CO, it should remain on the Cu_2_O(100) surface. The results suggest that the formation of active reaction sites and species on Cu_2_O are a result of the PY surface reconstruction on the Cu_2_O(111) surface.

**Figure 2:**
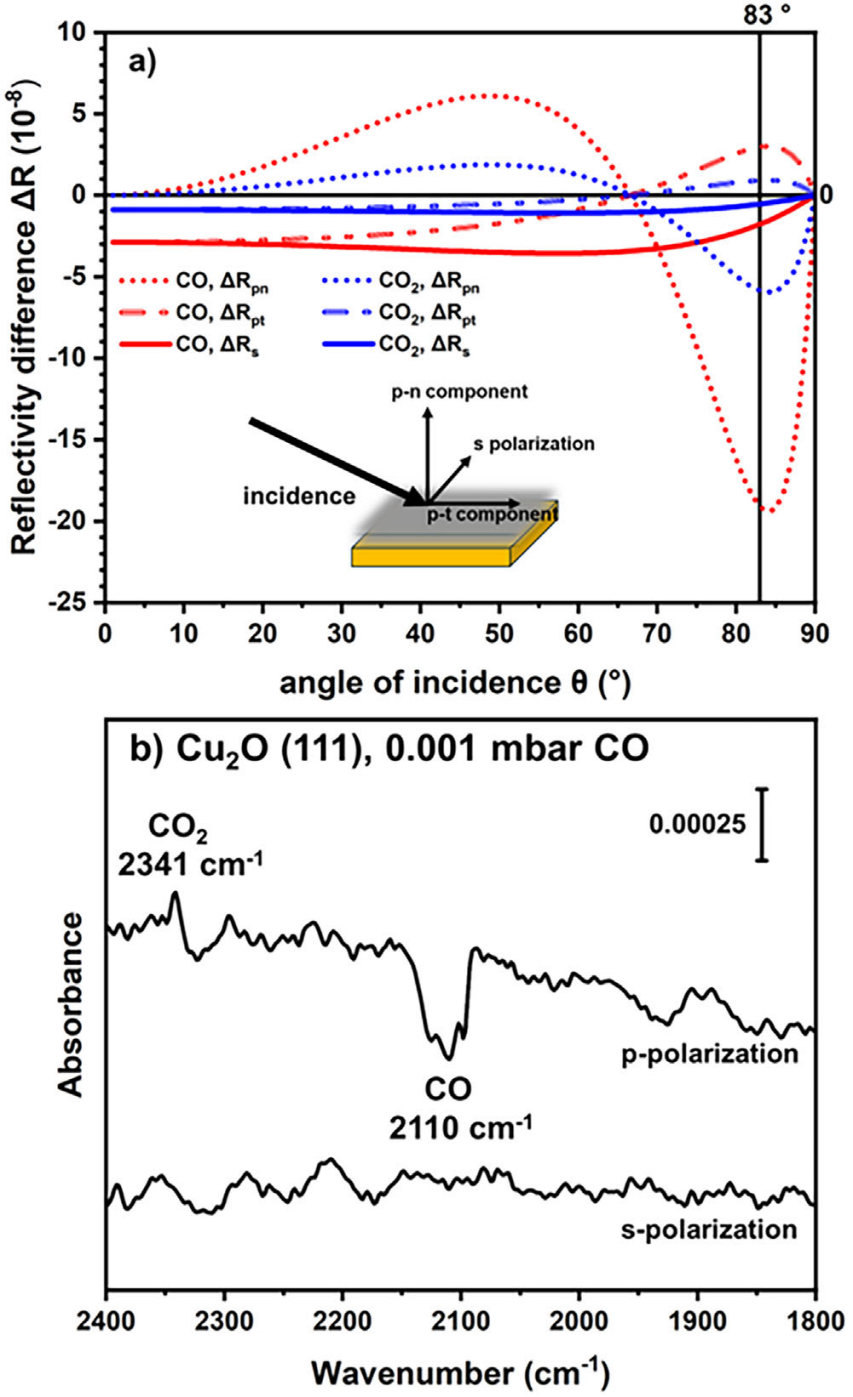
a) Calculated reflectivity differences (Δ*R* = *R*_0_–R) between the clean Cu_2_O(111) surface (*R*_0_) and the adsorbate‐covered Cu_2_O(111) surface (*R*) as a function of the angle of incidence (*θ*) are presented. The reflectivity is separated into s‐polarized light (s) and the normal (*p*_n_) and tangential (*p*_t_) components of p‐polarized light for the CO peak at 2110 cm^−1^ (red) and the CO_2_ peak at 2341 cm^−1^ (blue). The 83° angle of the experiment is marked. b) Absorbance spectra of surface species on a Cu_2_O(111) surface under 0.001 mbar CO show no peaks with s‐polarized light and a negative CO peak and positive CO_2_ peak in the p‐polarized spectrum. The data were collected at 140 K.

Based on vibrational frequencies in IRRAS, surface binding sites, chemical bonding, and orientation of adsorbates can be probed.^[^ ^23^ ^]^ While there is a large number of IRRAS studies on metals, only limited experimental IR data have been reported for single‐crystalline metal oxides because of their lower reflectivity.^[^ ^29^, ^30^, ^31^ ^]^ Further geometric details of the adsorbates can be gleaned from analysis of the sign of the adsorbate peaks collected at different light polarization, an advantage of using a semiconducting metal oxide single crystal as a substrate. Adsorbates with a molecular vibration component parallel to the s‐polarized light have a negative IR absorption signal; otherwise, there is no adsorbate signal in the spectrum collected with s‐polarization. For p‐polarized light, adsorbate vibrations have vector components normal and tangential to the substrate, and the vector magnitudes have opposite signs. The expected reflectivity changes of the normal and tangential components of the p‐polarized light were calculated for CO and CO_2_ vibrations and are presented in Figure 2a. The expected reflectivity difference (Δ*R*) can be compared to absorbance spectra found by plotting log(*R*/*R*_0_), where *R* is the reflectance of the surface under CO pressure and *R*_0_ is the reflectance of the clean surface. At the selected pressure of 0.001 mbar of CO in Figure 2b, gas phase contributions are insignificant. The lack of a CO feature in the s‐polarized data indicates that CO is not parallel to the surface. Furthermore, the negative signal of CO in the p‐polarized spectrum (Figure 2b) combined with the negative Δ*R* for the tangential p‐component in Figure 2a (red dots) indicate that the CO is completely or nearly perpendicular to the surface. For CO_2_, the tangential component of the p‐polarized light is positive (Figure 2a, blue dashes). Since the CO_2_ feature in Figure 2b is positive, we can conclude that the CO_2_ is not perpendicular to the surface and has a large tangential component in the adsorbate vibration (i.e., approaching a parallel orientation).

### Adsorbate Desorption Behavior

**Figure 3:**
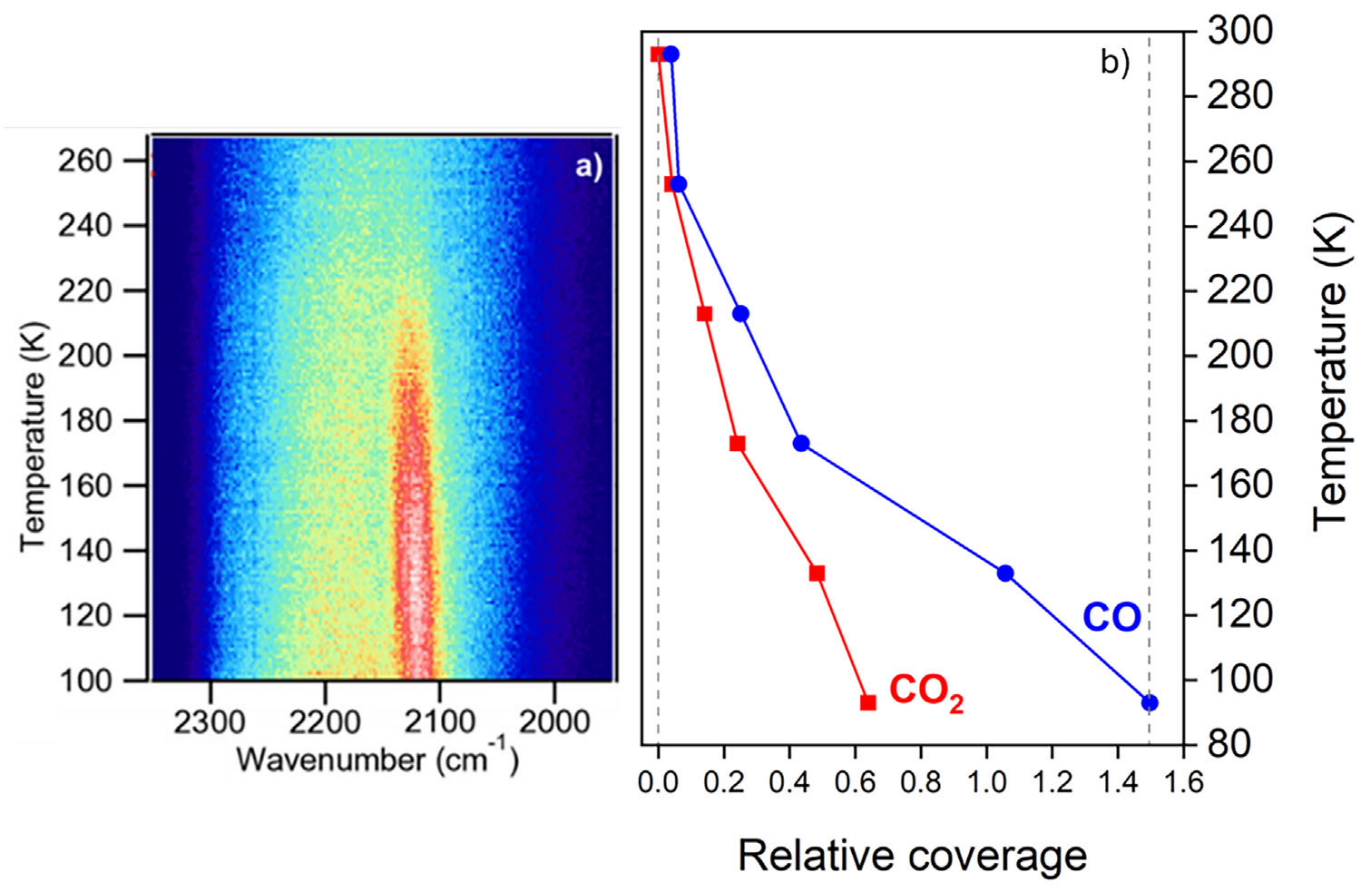
a) SFG spectra are plotted as a function of temperature, showing that CO fully desorbs by 230 K. b) Integrated C 1s peak areas from XPS data of CO (blue) and CO_2_ (red) components show adsorbates are removed below room temperature. The relative coverage is normalized to the estimated CO coverage of 1.5 molecules per surface unit cell (see Supporting Information).

A complementary molecular‐vibrational spectroscopy technique, sum‐frequency generation (SFG),^[^ ^32^, ^33^ ^]^ was used to confirm the adsorption of CO to the Cu_2_O(111) surface and interrogate its thermal stability. Figure 3a shows the spectra intensity plotted as a function of temperature beginning at 100 K. The CO vibration is the intense red feature. The CO feature begins to weaken around 150 K and disappears below room temperature, around 230 K. The CO_2_ vibration is outside the spectral range available in our SFG setup.

To quantify further the adsorption and reaction of CO on the reconstructed Cu_2_O(111) surface and study the desorption behavior of CO_2_, high resolution X‐ray photoelectron spectroscopy (XPS) data were collected after saturation of CO at cryogenic temperatures (93 K). From the components in the O 1s core level spectra, a CO*_x_* coverage of ∼3 oxygen atoms per surface unit cell is calculated (see Supporting Information). The C 1s core level spectra contain a main CO peak 288.1 eV and several corresponding shake‐up satellite peaks >290 eV (Figure S4); this binding energy and satellite position are consistent with CO adsorption on Cu_2_O films formed on Cu(111).^[^ ^34^ ^]^ Two minority peaks at lower binding energies (287.2 and 286.6 eV) are consistent with CO bound to more reduced and metallic Cu, respectively.^[^ ^34^, ^35^, ^36^ ^]^ A second peak in the C 1s spectrum at 289.0 eV matches literature reports of CO_2_ on copper surfaces.^[^ ^34^ ^]^ A small amount of adventitious carbon (<4% of the total surface carbon) is at 284.8 eV and can clearly be distinguished from the oxidized carbon peaks (Figures S4 and S5). The peak areas of all CO components and the CO_2_ component were used to calculate coverages (see Supporting Information), and these coverages are plotted in Figure 3b. CO desorbs between 213 K and 253 K; the 230 K desorption temperature from the SFG data in Figure 3a falls within this temperature range. The CO_2_ has completely desorbed before room temperature.

There is a small amount of potassium impurity (0.03 atoms per unit cell, see Supporting Information). The K peaks were held constant during the C 1s peak fitting and quantification. Surface K has been shown to facilitate carbonate formation under CO_2_ pressures and X‐ray irradiation. While the C 1s binding energy range for carbonates is 289.3–288.9 eV,^[^ ^37^, ^38^ ^]^ near‐edge X‐ray absorption fine structure (NEXAFS) spectroscopy data of the CO‐saturated surface lacks a distinct carbonate feature at 290.4 eV (Figure S7).^[^ ^39^ ^]^ Additionally, carbonates have been shown to remain on this surface until 500 K,^[^ ^38^ ^]^ so the complete removal of oxygenated carbon species in Figure 3b supports the absence of carbonate.

### Calculated Adsorption Energies and Geometries

**Figure 4:**
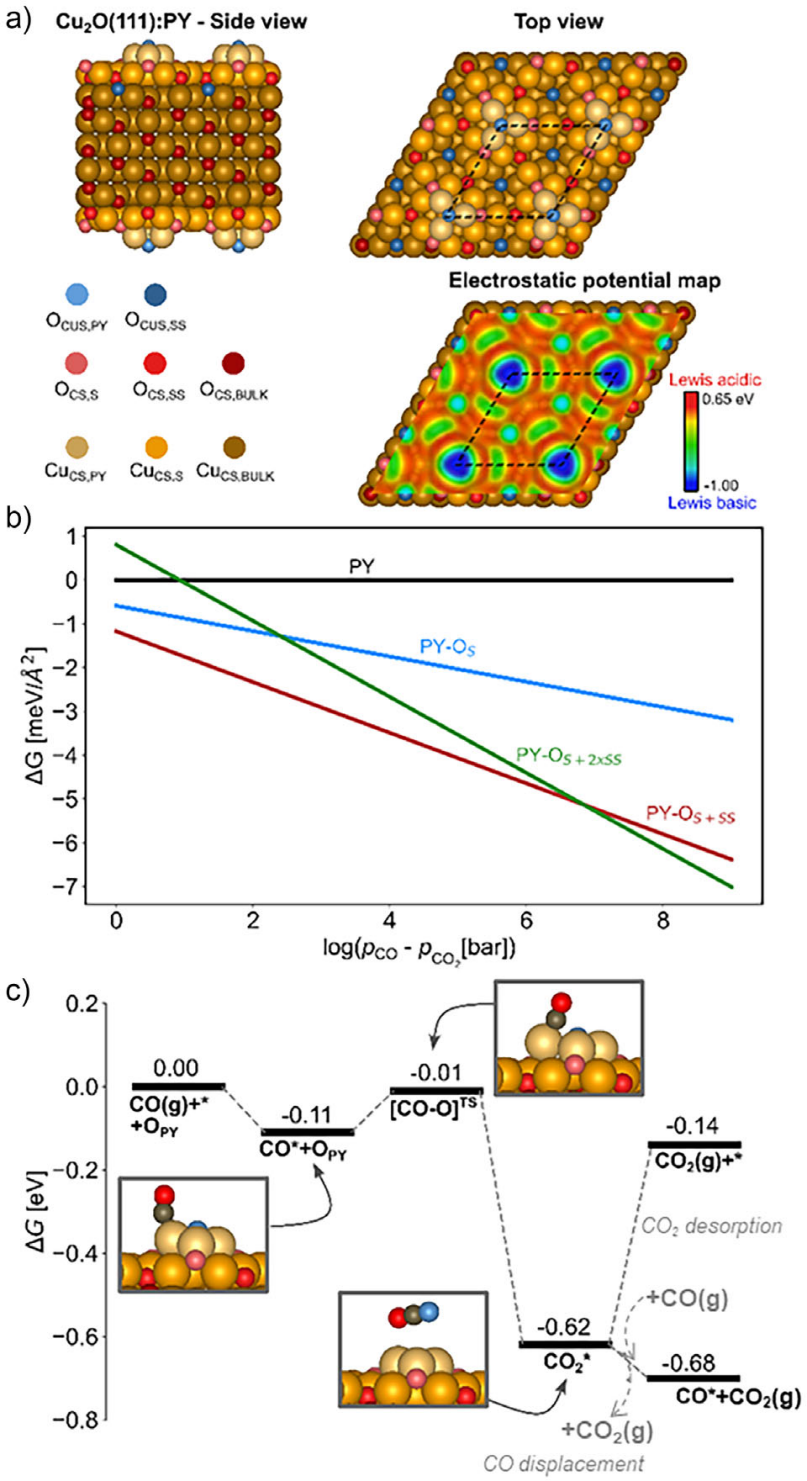
a) Atomic structure of the pyramidal (PY) (√3 × √3)*R30°* reconstruction structure of the Cu_2_O(111) surface, where subscript S indicates surface atom and SS indicates a subsurface atom. The electrostatic potential map is shown at the 0.001 a.u. isodensity contour, identifying Cu_CS,PY_ (O_CUS,PY_) as the most Lewis acidic (basic) sites. b) Relative stability of mildly CO‐reduced PY as a function of the pressure difference of CO(g) versus CO_2_(g) at 140 K. c) Mechanism for surface reduction by CO at 140 K and assuming partial pressures of *p*_CO _= 10^3^ *p*_CO2_ = 1 mbar. The final step indicates two possibilities: CO_2_ desorption or displacement of CO_2_ by CO. In C, the shortest C─Cu bond distances are (in Å) 1.93, 3.51, and 1.85 for CO@PY(stoichiometric), CO_2_@PY(V_O_), and CO@PY(V_O_), respectively.

Density functional theory (DFT) simulations of CO and CO_2_ interactions with the unreconstructed and PY reconstructed Cu_2_O(111) surfaces provide additional insight into the energetics of the CO adsorption and CO_2_ formation. Figure 4a shows that the naturally PY reconstructed surface prior to reduction exposes coordinatively saturated Cu, as well as saturated and unsaturated O atoms. The PY reconstructed surface is observed experimentally, as suggested from LEED and STM data (Figure S1) in comparison to Gloystein et al.^[^ ^11^, ^12^, ^13^ ^]^ The pyramidal Cu_4_O surface cluster contains coordinatively saturated Cu (Cu_CS,PY_) and coordinatively unsaturated O (O_CUS,PY_). Saturated surface (index S) and subsurface (index SS) O atoms and Cu atoms in slightly different coordination environments are the most common types of surface atoms. On the pristine surface, unsaturated O atoms are also exposed in the vacancies of unsaturated Cu (Cu_CUS_).

In comparison to the unreconstructed surface, the PY reconstructed surface displays weak susceptibility to interactions with CO. Whereas CO adsorbs strongly (Δ*G*_ad_ = −1.11 eV) at the Cu_CUS_ site of the unreconstructed Cu_2_O(111) surface, the strongest adsorption at the PY reconstructed surface is −0.1 to −0.2 eV at saturated Cu sites, including the Cu_CS,PY_ at the pyramidal Cu_4_O clusters and at a Cu_CS_ located at the vicinity of the cluster (see Table S1). However, exposure to CO is expected to lead to mild reduction of the PY reconstructed surface, even at low temperatures of <140 K used in the spectroscopy experiments, and to create undercoordinated Cu sites that are more reactive (Figure 4b). A mild Cu reduction is suggested further by a decrease in the calculated Cu Bader partial charge upon CO adsorption (see Table S2). The calculated CO vibrational frequencies also indicate slight Cu reduction. CO bound to the PY reconstructed surface has a calculated frequency of 2089 cm^−1^, and with an O_CUS,PY_ vacancy, the calculated CO frequency is 2103 cm^−1^. Both frequencies trend toward experimental values seen for CO bound to Cu^0^, which is consistent with the experimental frequencies at the lower range of expected CO bound to Cu^1+^ and with the C 1s XPS data showing CO bound to reduced Cu. Depending on the partial pressure difference between CO and CO_2_ (*p*_CO_ – *p*_CO2_), one‐third of a monolayer (ML, i.e., 1 ML is 3 sites per surface unit cell) to 1 ML of O surface vacancies can be generated. This occurs through reaction of O_CUS_ sites (while O_CS_ reduction is endergonic) with CO; an O vacancy coverage of two‐thirds of a ML is predicted for log(*p*_CO_ – *p*_CO2_) < 7. For larger pressure differences, a 1 ML coverage of O vacancies is predicted (Figure 4b).

Figure 4c contains DFT results demonstrating that surface reduction is also kinetically available at the 140 K and below, which was used for the experiments; adsorbed CO readily reacts with O_CUS,PY_ forming CO_2_ with a free energy barrier of 0.10 eV (Figure 4c), including a 0.02 eV entropic barrier. The reaction barrier changes insignificantly to 0.11 eV upon reduction of a full ML of O_CS_ sites. The formed CO_2_ remains adsorbed (physisorbed) in an adsorption mode nearly parallel to the reduced surface site, agreeing with the geometric analysis from the IRRAS data. Bader analysis suggests a weak charge transfer to the adsorbed CO_2_ of 0.03 e^−^. The barrier for desorbing CO_2_ (i.e., the free energy of desorption) is 0.48 eV at *p*_CO2_ of 1 x 10^−3^ mbar, which is thermally available at temperatures above 140 K. Replacing CO_2_ with CO at this O_CUS,PY_ vacancy site is exergonic by 0.06 eV and leads to CO chemisorbed perpendicular to the surface at a Cu atop position, consistent with the orientation found from the IRRAS and NEXAFS data (Figure S7). The calculated CO_2_ vibrational frequency, 2335 cm^−1^, is quite close to the experimental value of 2341 cm^−1^, further linking the computations with the experimental results.

The adsorption positions for CO can be rationalized by the variation in the surface electrostatic potential, *V*_S_(**r**), evaluated at an isodensity contour of the Cu_2_O(111) surface, as previously proposed by Stenlid et al.^[^ ^40^, ^41^ ^]^ For the unreduced PY reconstructed surface, the *V*_S_(**r**) displays maxima at the Cu_CS,PY_ and Cu_CS,S_ sites (Figure 4a), as well as at the unsaturated Cu_PY_ sites of the reduced surface (see Figure S8). These maxima correspond to Lewis acidic sites likely to interact with the electron lone pair on the C atom of the CO molecule, which rationalizes why these sites are the favored for CO interaction on the PY reconstructed surfaces. Adsorption energies for other surface terminations of Cu_2_O(111) are included in Table S1 for comparison.

## Conclusion

The formation of CO_2_ at cryogenic temperatures, below 100 K, on a metal oxide without needing a metal nanoparticle is striking. Our study shows that the oxygen atom at the Cu_4_O clusters of the PY surface reconstruction are highly reactive. The intrinsic reactivity of an oxygen atom on a surface reconstruction that is seen here stands in contrast to the peroxide intermediate required when oxidizing CO on ceria.^[^ ^8^, ^42^ ^]^ These results indicate that oxide surfaces can have highly reactive ensembles that could be dynamically formed during a catalytic reaction. In this case, cryogenic temperatures helped trap the formed CO_2_. Future studies will investigate the feasibility of regenerating the PY surface for a complete catalytic cycle. An understanding that some local surface ensembles of atoms are significantly more reactive than others will help guide material design to favor their formation.

## Supporting Information

The authors have cited additional references within the Supporting Information.^[^ ^43^, ^44^, ^45^, ^46^, ^47^, ^48^, ^49^, ^50^, ^51^, ^52^, ^53^, ^54^, ^55^, ^56^, ^57^, ^58^, ^59^, ^60^, ^61^, ^62^, ^63^, ^64^, ^65^, ^66^, ^67^, ^68^, ^69^, ^70^, ^71^, ^72^, ^73^, ^74^, ^75^, ^76^, ^77^, ^78^, ^79^, ^80^, ^81^, ^82^, ^83^, ^84^ ^]^

## Conflict of Interests

The authors declare no conflict of interest.

## Supporting information



Supporting Information

## Data Availability

The data that support the findings of this study are available in the Supporting Information of this article.
