# Supplementary material for: Cryogenic Carbon Monoxide Oxidation on Cuprous Oxide
Source: Angew Chem Int Ed Engl. 2025 Nov 10;65(1):e15673. doi: 10.1002/anie.202515673 (PMC12759210; doi:10.1002/anie.202515673)
Supplement: Supplementary file 1 — Supporting Information [file ANIE-65-e15673-s001.docx]

Supporting Information

Cryogenic Carbon Monoxide Oxidation Cuprous Oxide

Burcu Karagoz,^[a]†^ Tianhao Hu,^[b]†^ Joakim Halldin Stenlid,^[c, d, e, f]‡^ Xiaoming Hu,^[f]^ Markus Soldemo,^[e,f]^ Frank Abild-Pedersen,^[c]^ Kess Marks,^[e]^ Henrik Öström,^[e]^ Dario Stacchiola,*^[g]^ Jonas Weissenrieder,*^[g]^ Ashley R. Head,*^[g]^

[a] Diamond Light Source, Diamond House, Didcot, OX11 ODE, United Kingdom

[b] Department of Chemistry, Stony Brook University, Stony Brook, NY, 11974, United States

[c] SUNCAT Center for Interface Science and Catalysis, SLAC National Accelerator Laboratory, Menlo Park, CA, 94025, United States

[d] SUNCAT Center for Interface Science and Catalysis, Department of Chemical Engineering, Stanford University; Stanford, CA, 94305, United States

[e] Department of Physics, AlbaNova University Center, Stockholm University, Stockholm, SE-106 91, Sweden

[f] Light and Matter Physics, Applied Physics, KTH Royal Institute of Technology, Stockholm, SE-100 44, Sweden

[g] Center for Functional Nanomaterials, Brookhaven National Laboratory, Upton, NY 11973
† These authors contributed equally to this work

‡ Present address: Department of Chemistry and Chemical Engineering, Chalmers University of Technology, Gothenburg, SE-412 96, Sweden

**This PDF file includes:**

Materials

Infrared Reflection Absorption Spectroscopy

Reflectivity Difference Calculations

X-ray Photoelectron Spectroscopy

Estimation of Surface Adsorbate Coverage Using XPS

Assignment of XPS C 1s Species

Comments on the Potassium and Adventitious Carbon Contamination

NEXAFS Data Collection and Analysis

Sum Frequency Generation Vibrational Spectroscopy

Computational Methods

Additional Computational Results

References

**Experimental Section**

Materials

The Cu_2_O(111) and Cu_2_O(100) single crystals were purchased from Surface Preparation Laboratory, The Netherlands. Both Cu_2_O single crystals followed the same pretreatment procedure before each experiment.^[1]^ The Cu_2_O surface was cleaned by cycles of Ar^+^ sputtering (0.5 kV) for 20 min and annealing in O_2_ at 3 × 10^-6^ mbar at 600 °C for 10 min followed by annealing in UHV at 500 °C for 5 min. This treatment has been shown to give the PY surface reconstruction for Cu_2_O(111),^[2]^ and we have characterized this surface over many experiments ourselves.^[3–5]^ Fig. S1 shows a representative STM of the PY reconstructed surface.


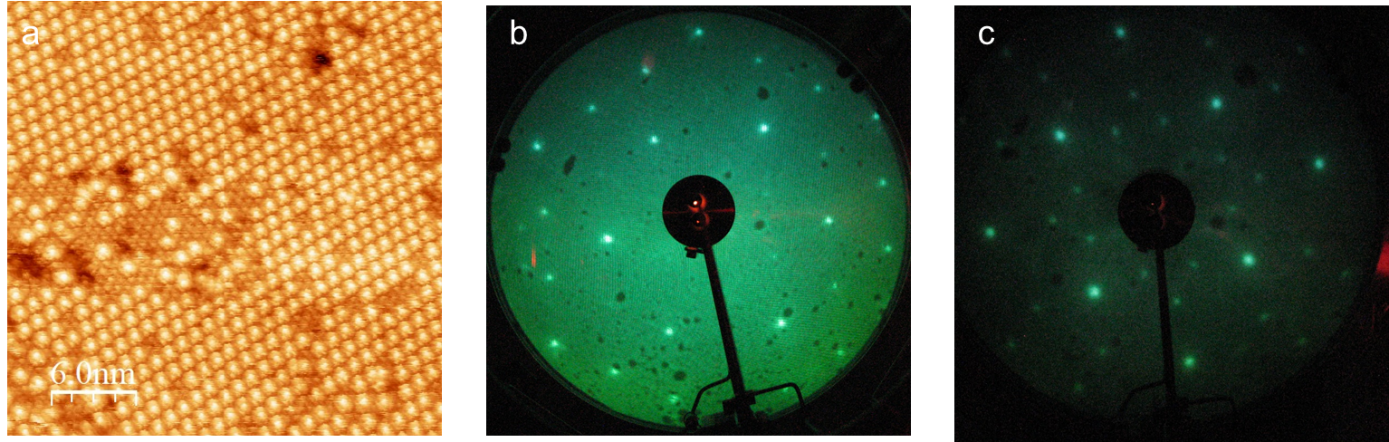


**Fig. S1.** A representative (a) scanning tunneling microscopy image and (b) LEED image (34.3 eV) of an as-prepared Cu_2_O(111)-(√3 × √3) surface. Minor regions of Cu_2_O(111)-(1 × 1) termination can be observed in the STM image. The STM image dimensions are 30 nm x 30 nm, and the scanning parameters are -1.0 V and 0.05 nA. (c) A representative LEED image (44.6 eV) of the Cu_2_O (100) surface.

Infrared Reflection Absorption Spectroscopy

IRRAS experiments were performed in Brookhaven National Laboratory using a Bruker 80v spectrometer.^[6]^ IR light exits the spectrometer, travels through a wire grid polarizer, enters an ultra-high vacuum (UHV) chamber through a KBr window, is reflected off the sample, exits the chamber through a second KBr window, and is detected with a liquid-nitrogen cooled mercury-cadmium-telluride detector. The base pressure of the IRRAS chamber is better than 5 x 10^-9^ mbar. The spectrometer optics and detector are under a rough vacuum. The sample was cooled in the range of 115 K to 140 K under all conditions. Spectra were collected under UHV conditions and under increasing pressures of CO with both p- and s- polarized light. The spectra are an average of 10,000 scans (40 min collection time) and have 4 cm^−1^ resolution. The cleanliness of the sample was verified with in situ XPS prior to IRRAS experiments.

Reflectivity Difference Calculations

Starting with the equation for reflectance and absorbance, $\Delta A\equiv{log}_{10}(R^{0}/R)$, reflectivity difference of the Cu_2_O surface before ($R^{0}$) and after ($R$) CO or CO_2_ adsorption as a function of the incidence angle ($\theta$) was calculated as follows:^[7–9]^

$$\Delta R_{i}\left( \theta\right)=R_{i}^{0}\left( \theta\right)- R_{i}\left( \theta\right)= R_{i}^{0}\left( \theta\right)\left( 1 -\frac{1}{{10}^{\Delta A_{i}\left( \theta\right)}} \right)$$

in which, $i$ represents each component of polarized light and $R^{0}$ can be computed by the following equations.

$$R_{s}^{0}\left( \Theta\right)=\left( \left| \frac{\xi_{3}\left( \theta\right)- \xi_{1}\left( \theta\right)}{\xi_{3}\left( \theta\right)+ \xi_{1}\left( \theta\right)} \right| \right)^{2}$$

$$R_{pt}^{0}\left( \theta\right)= R_{pn}^{0}\left( \theta\right)= \left( \left| \frac{\frac{\xi_{3}\left( \theta\right)}{{n_{3}}^{2}}-\frac{\xi_{1}\left( \theta\right)}{{n_{1}}^{2}}}{\frac{\xi_{3}\left( \theta\right)}{{n_{3}}^{2}}+\frac{\xi_{1}\left( \theta\right)}{{n_{1}}^{2}}} \right| \right)^{2}$$

where

$$\xi_{j}\left( \theta\right)= \surd[{\hat{n}_{j}}^{2}- n_{1}\cdot\left( \sin\theta\right)^{2}]$$

with the complex refractive index $\hat{n}$ where $n$ is the refraction index and $k$ is the extinction coefficient

$$\hat{n}_{j}=n_{j}+i\cdot k_{j}$$

and the absorbance $\Delta A$

$$\Delta A_{x}\left( \theta\right)=\left( -\frac{16\pi}{ln10} \right)\cdot\left( \frac{cos\Theta}{\left( \frac{\xi_{3}\left( \theta\right)^{2}}{{n_{3}}^{4}} \right)-\left( cos\theta\right)^{2}} \right)\cdot\left( \frac{-\left( \xi_{3}\left( \theta\right) \right)^{2}}{{n_{3}}^{4}} \right)\cdot\left( \frac{n_{2}\cdot k_{2}\cdot d_{2}}{\lambda} \right)$$

$$\Delta A_{y}\left( \theta\right)=\left( -\frac{16\pi}{ln10} \right)\cdot\left( \frac{cos\theta}{{n_{3}}^{2}-1} \right)\cdot\left( \frac{n_{2}\cdot k_{2}\cdot d_{2}}{\lambda} \right)$$

$$\Delta A_{z}\left( \theta\right)=\left( -\frac{16\pi}{ln10} \right)\cdot\left( \frac{cos\theta}{\left( \frac{\xi_{3}\left( \theta\right)^{2}}{{n_{3}}^{4}} \right)-\left( cos\theta\right)^{2}} \right)\cdot\left( \frac{\left( sin\theta\right)^{2}}{\left( {n_{2}}^{2}+{k_{2}}^{2} \right)^{2}} \right)\cdot\left( \frac{n_{2}\cdot k_{2}\cdot d_{2}}{\lambda} \right)$$

The $x$ and $z$ subscripts refer to tangential (pt) and normal (pn) components of p polarization, respectively. The $y$ subscript corresponds to s polarization. $d_{2}$ is the thickness of the adsorption layer and $\lambda$ is the wavelength of the respective light.

The following values of optical parameters are used in calculations: n_1_(vacuum) = 1, k_1_ = 0, n_2_(CO) = 1.0003^[10]^, k_2_(CO) = 0.000655,^[11]^ d_2_(CO) = 0.115 nm,^[12]^ n_2_(CO_2_) = 1.0004,^[13]^ k_2_(CO_2_) = 0.0001,^[9]^ d_2_(CO_2_) = 0.208 nm,^[14,15]^ n_3_(Cu_2_O) = 2.310,^[16]^ k_3_ = 0.042.^[16]^

**
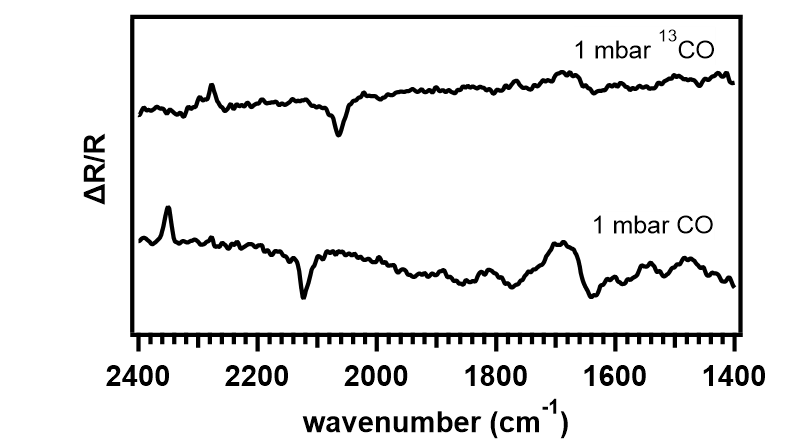
**

**Fig. S****2.** An expanded wavenumber range of the IR data of Cu_2_O under 1 mbar of ^13^CO (top) and 1 mbar CO (bottom) shows no obvious carbonates around the expected region of 1600 to 1400 cm^-1^.

X-ray Photoelectron Spectroscopy

The XPS measurements were conducted at the surface science high-resolution photoelectron spectroscopy end stations of the FlexPES^[17]^ and I311^[18]^ beamlines at MAX IV, Sweden. The analysis chambers of the end stations were equipped with a Scienta Omicron DA30-L and SES200 analyzers, respectively. The preparation chambers, in direct connection with the analysis chambers, were equipped with argon ion sputter guns, low energy electron diffraction optics, and precision leak-valves for gas dosing. The base pressures in both chambers were 1 × 10^-10^ mbar. The Cu_2_O(111) sample was mounted on a stainless-steel sample plate and the temperature was monitored using a K-type thermocouple spot-welded in proximity to the sample. The sample holder was mounted on a liquid nitrogen cryostat. The sample was prepared based on literature procedures previously demonstrated to give the (111) PY reconstructed surface

Estimation of Surface Adsorbate Coverage Using XPS

The inelastic mean free path (λ_Cu2O_) of electrons with 169 eV kinetic energy propagating in Cu_2_O is 6.1 Å according to the predictive model in ref [42]. In calculating λ_Cu2O_ we use the following parameters for Cu_2_O: band gap 2.2 eV, density 6 g/cm^3^, 11 valence electrons for Cu, and 6 valence electrons for O. The O-O plane distance in the (111) direction of Cu_2_O is 1.23 Å. From the l_Cu2O_ and the atomic structure of Cu_2_O we calculate the intensity contribution from the O atoms residing at the surface of a bulk truncated O terminated clean Cu_2_O(111) surface (Fig. S3(a)) to 18.3% of the total O 1s intensity.

We estimate the CO_x_ coverage from its XPS O 1s intensity contribution in Fig. S3(b) to the total O 1s intensity. The CO_x_ contribution to the total O 1s intensity is 19% (after subtraction of overlapping Cu_2_O photoemission contribution). This translates to 3 oxygen atoms per surface unit cell. The CO C 1s peak intensities (including satellites) are twice the CO_2_ C 1s intensity (see Figure S4 below). The combined C 1s and O 1s information allows us to estimate an average coverage of ~0.75 CO_2_ molecules per surface unit cell (at the PY site) and ~1.5 CO molecules per surface unit cell.


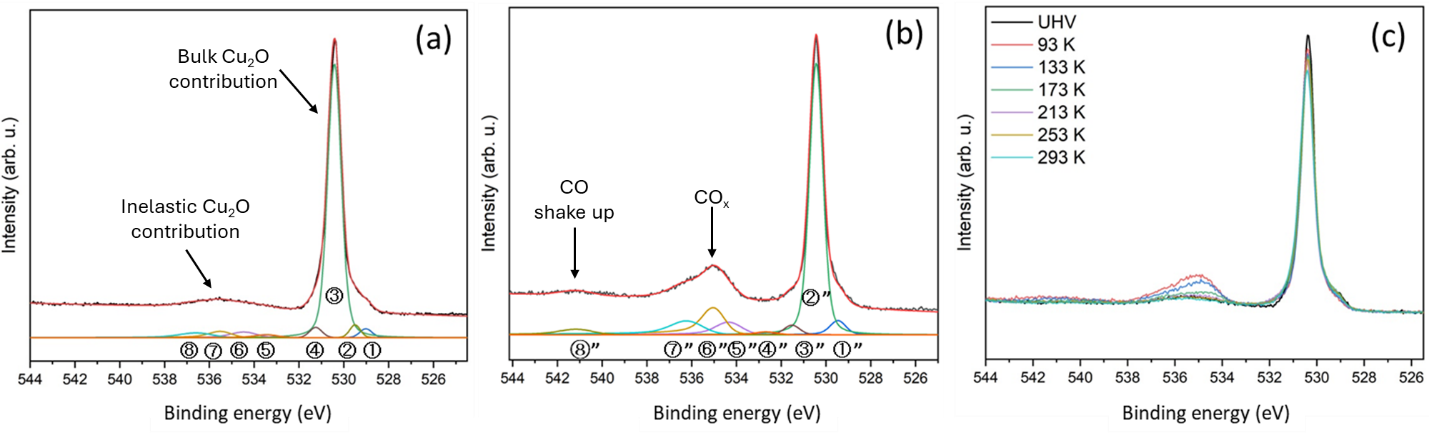


**Fig. S3.** XPS O 1s core level spectra (hν = 700 eV) collected at 93 K. **(a)** clean reconstructed Cu_2_O(111) surface, **(b)** after a 2.5 L dose of CO, and **(c)** at increasing temperature heating to the temperatures listed.


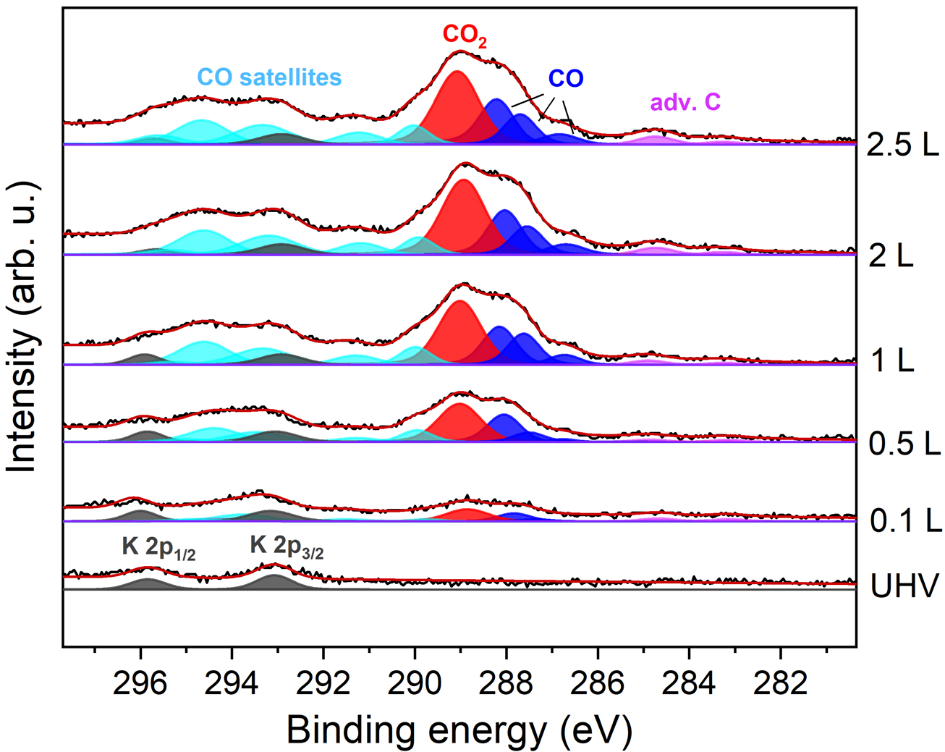


**Fig. S4.** Background subtracted C 1s spectra (hν = 380 eV) from the Cu_2_O(111) surface after increasing doses of CO in units of Langmuir (L) at 93 K.

Assignment of XPS C 1s Species

Fig. S4 shows the evolution of the XPS C 1s region with increasing CO doses. The as-prepared clean surface (UHV) exhibits a trace of potassium, likely originating from K impurities in the bulk of the Cu_2_O crystal. The K 2p spin-orbit split peak areas (at 293.05 eV and 295.95 eV) were held constant in all spectral fits. We assign the peaks at 288.1 eV, 287.6 eV, and 286.7 eV to CO adsorbate. The peak at 289.0 eV is assigned to CO_2_ at the PY site, after reaction with the site’s oxygen atom. Remaining peaks at binding energies >289 eV are assigned to CO shake-up satellites. Note: although the peak at 291.3 eV is at a binding energy similar to physisorbed CO_2_ (spectrum CO_2_ in Fig. S6) its desorption characteristics follows CO, while physisorbed CO_2_ is found to desorb already <100 K. The relative C 1s intensity of CO (including satellites) to CO_2_ is approximately 2:1.

Comments on the Potassium and Adventitious Carbon Contamination

The relative spectral abundance of K compared to the CO_x_ species on the surface can be estimated from the K 2p and C 1s integrated intensities after considering the photoionization cross sections for K 2p (2.0160 Mbarn) and C 1s (0.5251 Mbarn).^[20]^ This results in a relative concentration of 1.56% (cross section adjusted K 2p intensity / cross section adjusted C 1s intensity from CO_x_ lines and satellites) for the 2.5 L CO spectrum. Or **~**0.03 K atoms per surface (√3x√3)30° unit cell. Carbonate species are known to form on Cu_2_O and other metal oxides when potassium is present,^[21,22]^ and carbonate has an expected binding energy at 289.3 eV.^[23]^ However, there is no evidence of carbonate in the NEXAFS (Fig. S7) or IRRAS data (Fig. S2), as mentioned in the main manuscript.

We observe traces of adventitious carbon (adv. C) that grows with instrument time (3.89% compared to the CO_x_ species in the 2.5 L CO spectrum). The adventitious carbon is a result of adsorption from the residual gas in the analysis chamber.


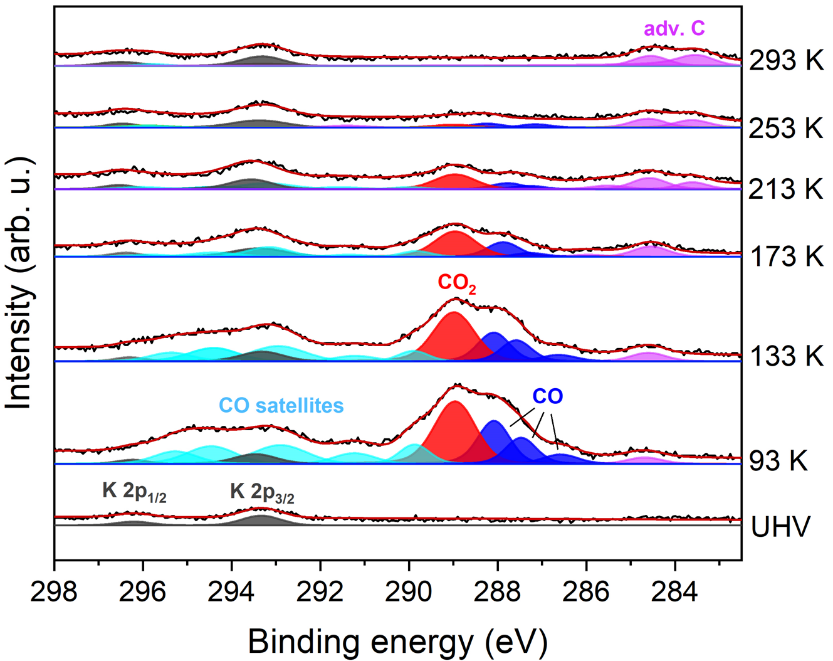


**Fig. S5.** Background subtracted C 1s spectra (hν = 380 eV) of the reconstructed Cu_2_O(111) surface before (UHV) and after a saturation dose (2.5 L) of CO at 93 K as well as at incrementally increased sample temperatures. All spectra were collected at the indicated temperatures to avoid potential re-adsorption. The fitted C 1s peak intensities of the CO (including satellites) and CO_2_ components were extracted to Fig. 3b of the main manuscript.


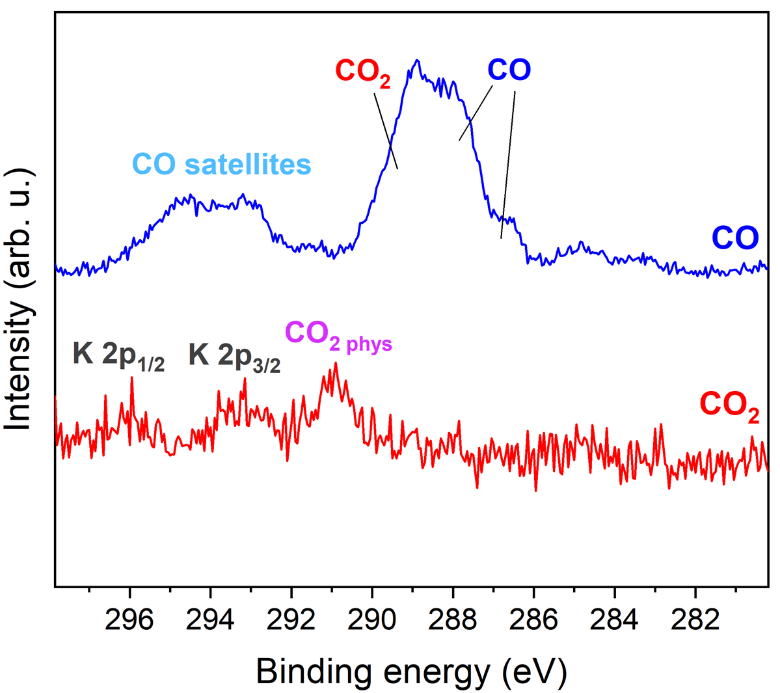


**Fig. S6.** XPS C 1s spectra (hν = 380 eV) from Cu_2_O(111) at 93 K. The spectrum labeled CO_2_ (red) was collected under a CO_2_ total pressure of 5 × 10^-9^ mbar to stabilize physisorbed CO_2_ since desorption of CO_2_ was observed already at 93 K under UHV conditions. The peak at 291.2 eV is assigned to physisorbed CO_2_ on sites that have not been reduced by CO adsorption. The spectrum labeled CO (blue) was collected after dosing of 1L CO at 93 K.

NEXAFS Data Collection and Analysis

Near-edge X-ray absorption fine structure data was collected at B07B ES-1 at the Diamond Light Source, Didcot, UK ^[24]^. The data were collected in Auger electron yield mode with the photon beam angle set to 0° and 60° relative to the surface normal. The spectra covered a kinetic energy range of 255 eV to 280 eV, capturing the carbon KLL Auger emission, while the photon energy was scanned from 278 to 310 eV in 0.2 eV steps. Measurements were taken for both the clean surface and after CO adsorption at 140 K. The 2D spectra were normalized by the ring current and emitted photoelectrons, using polished copper plates facing the two horizontal optical mirrors. The data shown below were integrated over a narrower kinetic energy range (260 eV to 272 eV) to eliminate artificial features caused by photoemission peaks, and the clean-surface spectra were subtracted after undergoing the same treatment. Finally, the spectra were normalized to the signal at 300 eV in high photon energy. The surface was saturated with CO via dosing ~5 × 10^-8^ mbar CO for several minutes in a preparation chamber; saturation coverage was confirmed by XPS. NEXAFS data were collected in the analysis chamber in a pressure better than ~1 × 10^-8^ mbar, with the background pressure being residual CO.

**Fig. S7.** C K-edge NEXAFS data of CO adsorbed on to the reconstructed Cu_2_O(111) surface at 140 K with an incident photon angle of 0° and 60° relative to the surface normal. The sharp peak at 288.1 eV is consistent with adsorbed CO and CO_2_. There is no carbonate peak at 290.4 eV.

Sum Frequency Generation Vibrational Spectroscopy

The SFG experiments were performed using a surface science instrument at Stockholm University. The laser light was produced using a Ti:Sa amplifier femtosecond laser, providing pulses at 800 nm with a pulse duration of 37 fs, repetition rate of 1 kHz and a pulse energy of 4 mJ. The IR light entered the UHV chamber via a BaF_2_ viewport and the SFG light exited via a CaF_2_ viewport. The remaining 800 nm light was spectrally filtered using a 750 nm short wave pass filter. The SFG was spectrally analyzed and detected using an optical spectrograph, equipped with an ICCD camera allowing simultaneous detection of the entire probed spectral region for each laser shot. The base pressure of the chamber was 5 x 10^-10^ mbar, and sample cleanliness was verified using LEED and SFG prior to experiments. The sample was mounted on a liquid nitrogen cryostat and heated at a constant rate of 3 K/min during the experiment to observe the desorption kinetics.

Computational Methods

Symmetric Cu_2_O(111) surface slab models were generated from an optimized (cubic) bulk structure with a lattice parameter of 4.29 Å, which is in close agreement with experimental reports of 4.27 Å.^[25–27]^ The models comprise seven Cu_2_O layers with, the two top and bottom layers free to relax and the remaining atoms fixed at their lattice positions. Stoichiometric as well as Cu or O deficient surfaces where considered, as specified in the figures and tables below. The (√3 × √3)*R30*° surface reconstruction (PY) with Cu_CUS_ vacancies and a pyramidal Cu_4_O cluster attached at one of the Cu_CUS_ vacancies was recently identified as the most stable surface termination of Cu_2_O(111) at the synthesis conditions applied herein ^[2,28]^. Therefore, the PY surface reconstruction is the primary surface termination considered herein with complementary data for other surface terminations found in the Fig. S8.

The surfaces were modeled using periodic spin-polarized density functional theory (DFT) calculations with the Vienna Ab initio Simulation Package (VASP)^[29–31]^ and the PBE functional^[32]^ with D3(BJ) dispersion corrections.^[33,34]^ Hubbard corrections^[35]^ were employed using a *U*-*j* value of 3.6 eV applied to the *d*-states of Cu, as suggested by Yu et al.^[36]^ A plane wave basis set with a cutoff of 600 eV was used for the valence states (Cu: 3d^10^4s^1^; O: 2s^2^2p^4^; C: 2s^2^2p^2^), and standard PBE PAW (projector augmented wave) potentials^[37,38]^ for the core states. A Γ-centered 3×3×1 k-point mesh was used for sampling of the Brillouin zone, and a 0.05 eV Gaussian electronic smearing. A minimum vacuum separation of 15Å was employed to avoid spurious interactions between surface slabs.

Thermal corrections were added from thermochemical analysis based on vibrational (surface and gas states), rotational (gas states only), and translational (gas) contributions from numerical frequency calculations using ±0.015 Å displacements analyzed using the Vaspkit code.^[39]^ The analysis is based on the harmonic oscillator, rigid rotor, and ideal gas approximations. Energy corrections of 0.01 and 0.65 eV were added to CO(g) and CO_2_(g) to ensure that gas-phase reaction energies are reproduced^[40,41]^ These corrections are based on established values for the PBE functional ^[41]^ and were aligned versus measured adsorption energies of CO on Cu(111)^[42–44]^ and Cu_2_O(100).^[45]^ Barriers were computed using the climbing image nudged elastic band (CI-NEB) method^[46]^ with nine intermediate images on smaller asymmetric surface models (including four Cu_2_O layers) and a plane wave cutoff of 400 eV.

Reactivity evaluation via the use of surface electrostatic potential maps, *V*_S_(r), was carried out as proposed and detailed by Stenlid and Brinck,^[47–49]^ aided by the VESTA software.^[50]^ Bader partial charges were computed using analysis scripts from the Henkelman and co-workers.^[51]^ Bader charges were obtained as deviations from the valence electron count of the corresponding neutral atoms defined by the PAW potentials.

Optimized structures, with corresponding energies, have been uploaded to the Catalysis Hub database^[52]^ and can be accessed directly via [https://www.catalysis-hub.org/publications/KaragozCryogenic2025](https://urldefense.com/v3/__https:/www.catalysis-hub.org/publications/KaragozCryogenic2025__;!!P4SdNyxKAPE!FWfgsTbOyDmNUns_mO07svGH2vF-Hg9qxg2VT_e_2bR8cygYlP3nbu6BrMFiBSMtR4vqAVv_rcrLhcoc$).

Additional Computational Results

Additional computational results regarding CO and CO_2_ adsorption onto various terminations of Cu_2_O(111) are included in Fig. S8 and Tables S1 and S2 below. Fig. S8 also includes surface electrostatic potential maps indicating sites susceptible to interactions with CO.


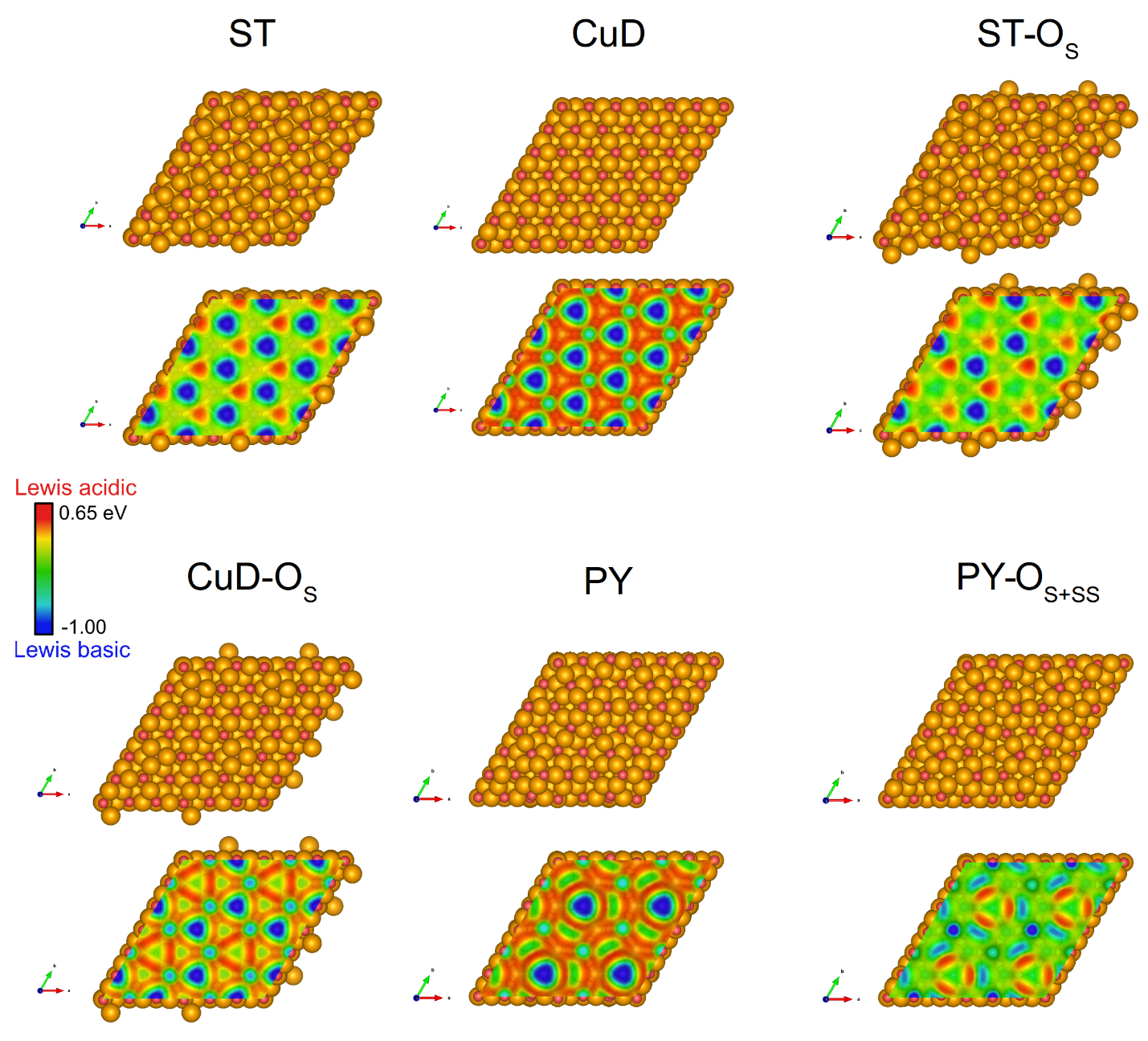


**Fig. S8.** Surface structures and corresponding 0.001 a.u. electrostatic potential maps of different surface terminations of Cu_2_O(111). ST = stochiometric, pristine; CuD = ST with 1ML Cu_CUS_ vacancies; ST-O_S_ = ST with ⅓ ML O_CUS_ vacancies; CuD-O_S_ = CuD with ⅓ ML O_CUS_ vacancies; PY = pyramidal reconstruction, i.e., CuD with ⅓ ML of Cu_4_O cluster adsorbed onto the Cu_CUS_ vacancies; PY-O_S+SS_ = PY with 1 ML O_CUS,PY_ and ⅓ ML of O_CUS,SS_ vacancies. Color code atomistic model: Cu(orange), O(red).

**Table S1**

The CO and CO_2_ adsorption energies onto the surface terminations of Cu_2_O(111) displayed in Figure S8. At 140 K and *p*_CO_=10^3^·*p*_CO2_=1 mbar. In the table, ‘vac’ indicates a vacancy.

| **Surface** | **Site** | **Δ*G*_ad_(CO), eV** | **Δ*G*_ad_(CO_2_), eV** |
| --- | --- | --- | --- |
| **PY** | Cu_CAS_ surface | -0.16 |  |
|  | Cu_CAS_ pyramid | -0.11 |  |
|  | Cu_CAS_ bridge long | -0.19 |  |
| **PY (reduced)** | O_py_-vac | -0.54 | -0.48 |
|  | O-vac Cu_CUS_-vac | -0.55 | -0.45 |
|  | O_PY_ |  | -0.45 |
| **ST** | Cu_CUS_ | -1.11 | -0.47 |
|  | Cu_CAS_ | 0.15 |  |
| **CuD** | Cu-vac bridge | 0.16 | -0.46 |
| **ST-O_S_** | Cu_CUS_ | -1.07 | -0.40 |
|  | O-vac bridge | -0.91 |  |
| **CuD-O_S_** | O-vac hollow | -1.10 | -0.15 |

**Table S2**

Calculated Bader charge (*q*) on the Cu atoms of different surfaces. * indicates adsorbed species.

| **Surface** | ***q* (Cu w/ CO_(2)_)** | ***q* (Cu w/o CO)** |
| --- | --- | --- |
| PY | n/a | 0.53 |
| PY (CO*) | 0.60 | 0.53 |
| PY:O-vac | n/a | 0.22 |
| PY:O-vac (CO*) | 0.38 | 0.21 |
| PY:O-vac (CO_2_*) | 0.23^a^ | n/a |

^a^ CO_2_ sits horizontally and interacts with 3 Cu atoms, giving a *q* ranging from 0.18 to 0.26 for Cu. The reported value is the average.

**References**

[1] A. Gloystein, J. A. Creed, N. Nilius, “Atomic View on the (111) Surface of a Cu_2_O Single Crystal: Reconstruction, Electronic Properties, and Band-Bending Effects” *J. Phys. Chem. C* **2022**, *126*, 16834–16840.

[2] A. Gloystein, N. Nilius, J. Goniakowski, C. Noguera, “Nanopyramidal Reconstruction of Cu_2_O(111): A Long-Standing Surface Puzzle Solved by STM and DFT” *J. Phys. Chem. C* **2020**, *124*, 26937–26943.

[3] M. Soldemo, J. H. Stenlid, Z. Besharat, M. Ghadami Yazdi, A. Önsten, C. Leygraf, M. Göthelid, T. Brinck, J. Weissenrieder, “The Surface Structure of Cu_2_O(100)” *J. Phys. Chem. C* **2016**, *120*, 4373–4381.

[4] H. Tissot, C. Wang, J. H. Stenlid, T. Brinck, J. Weissenrieder, “The Surface Structure of Cu_2_O(100): Nature of Defects” *J. Phys. Chem. C* **2019**, *123*, 7696–7704.

[5] C. Wang, Y. Kong, M. Soldemo, Z. Wu, H. Tissot, B. Karagoz, K. Marks, J. H. Stenlid, A. Shavorskiy, E. Kokkonen, S. Kaya, D. J. Stacchiola, J. Weissenrieder, “Stabilization of Cu_2_O through Site-Selective Formation of a Co_1_Cu Hybrid Single-Atom Catalyst” *Chem. Mater.* **2022**, *34*, 2313–2320.

[6] C. N. Eads, J.-Q. Zhong, D. Kim, N. Akter, Z. Chen, A. M. Norton, V. Lee, J. A. Kelber, M. Tsapatsis, J. A. Boscoboinik, J. T. Sadowski, P. Zahl, X. Tong, D. J. Stacchiola, A. R. Head, S. A. Tenney, “Multi-modal surface analysis of porous films under operando conditions” *AIP Adv.* **2020**, *10*, 085109.

[7] W. N. Hansen, “Reflection spectroscopy of adsorbed layers” *Symp. Faraday Soc.* **1970**, *4*, 27–35.

[8] J. A. Mielczarski, R. H. Yoon, “Fourier transform infrared external reflection study of molecular orientation in spontaneously adsorbed layers on low-absorption substrates” *J. Phys. Chem.* **1989**, *93*, 2034–2038.

[9] M. Buchholz, P. G. Weidler, F. Bebensee, A. Nefedov, C. Wöll, “Carbon dioxide adsorption on a ZnO(100) substrate studied by infrared reflection absorption spectroscopy” *Phys. Chem. Chem. Phys.* **2013**, *16*, 1672–1678.

[10] P. L. Smith, M. C. E. Huber, W. H. Parkinson, “Refractivities of H_2_, He, O_2_, CO, and Kr for 168≤λ≤288 nm” *Phys. Rev. A* **1976**, *13*, 1422–1434.

[11] K. M. Bulanin, A. Yu. Mikheleva, D. N. Shchepkin, A. V. Rudakova, “Determination of the Extinction Coefficient of Carbon Monoxide Adsorbed on Titanium Dioxide” *Opt. Spectrosc.* **2022**, *130*, 248–256.

[12] B.-Z. Sun, W.-K. Chen, Y.-J. Xu, “Coadsorption of CO and NO on the Cu_2_O(111) surface: A periodic density functional theory study” *J. Chem. Phys.* **2009**, *131*, 174503.

[13] A. Bideau-Mehu, Y. Guern, R. Abjean, A. Johannin-Gilles, “Interferometric determination of the refractive index of carbon dioxide in the ultraviolet region” *Opt. Commun.* **1973**, *9*, 432–434.

[14] H. Wu, N. Zhang, H. Wang, S. Hong, “Adsorption of CO_2_ on Cu_2_O(111) oxygen-vacancy surface: First-principles study” *Chem. Phys. Lett.* **2013**, *568–569*, 84–89.

[15] L. I. Bendavid, E. A. Carter, “CO_2_ Adsorption on Cu_2_O(111): A DFT+U and DFT-D Study” *J. Phys. Chem. C* **2013**, *117*, 26048–26059.

[16], M.R. Querry, "Optical constants" (ADA158623, U.S. Army Armament, Munitions, and Chemical Command, 1985). https://apps.dtic.mil/sti/citations/ADA158623

[17] A. Preobrajenski, A. Generalov, G. Öhrwall, M. Tchaplyguine, H. Tarawneh, S. Appelfeller, E. Frampton, N. Walsh, “FlexPES: a versatile soft X-ray beamline at MAX IV Laboratory” *J. Synchrotron Radiat.* **2023**, *30*, 831–840.

[18] R. Nyholm, J. N. Andersen, U. Johansson, B. N. Jensen, I. Lindau, “Beamline I311 at MAX-LAB: a VUV/soft X-ray undulator beamline for high resolution electron spectroscopy” *Nucl. Instrum. Methods Phys. Res. Sect. Accel. Spectrometers Detect. Assoc. Equip.* **2001**, *467–468*, 520–524.

[19] C. J. Powell, A. Jablonski, , NIST Electron Inelastic-Mean-Free-Path Database 71, Version 1.1. Nat'l Std. Ref. Data Series (NIST NSRDS) -, National Institute of Standards and Technology, Gaithersburg, MD (Accessed January 6, 2025).

[20] J. J. Yeh, I. Lindau, “Atomic subshell photoionization cross sections and asymmetry parameters: 1 ⩽ *Z* ⩽ 103” *At. Data Nucl. Data Tables* **1985**, *32*, 1–155.

[21] C. Wang, H. Tissot, C. Escudero, V. Pérez-Dieste, D. Stacchiola, J. Weissenrieder, “Redox Properties of Cu_2_O(100) and (111) Surfaces” *J. Phys. Chem. C* **2018**, *122*, 28684–28691.

[22] O. Rosseler, M. Sleiman, V. N. Montesinos, A. Shavorskiy, V. Keller, N. Keller, M. I. Litter, H. Bluhm, M. Salmeron, H. Destaillats, “Chemistry of NO_x_ on TiO_2_ Surfaces Studied by Ambient Pressure XPS: Products, Effect of UV Irradiation, Water, and Coadsorbed K+” *J. Phys. Chem. Lett.* **2013**, *4*, 536–541.

[23] X. Deng, A. Verdaguer, T. Herranz, C. Weis, H. Bluhm, M. Salmeron, “Surface Chemistry of Cu in the Presence of CO_2_ and H_2_O” *Langmuir* **2008**, *24*, 9474–9478.

[24] D. C. Grinter, P. Ferrer, F. Venturini, M. A. van Spronsen, A. I. Large, S. Kumar, M. Jaugstetter, A. Iordachescu, A. Watts, S. L. M. Schroeder, A. Kroner, F. Grillo, S. M. Francis, P. B. Webb, M. Hand, A. Walters, M. Hillman, G. Held, “VerSoX B07-B: a high-throughput XPS and ambient pressure NEXAFS beamline at Diamond Light Source” *J. Synchrotron Radiat.* **2024**, *31*, 578–589.

[25] A. Werner, H. D. Hochheimer, “High-pressure x-ray study of Cu_2_O and Ag_2_O” *Phys. Rev. B* **1982**, *25*, 5929–5934.

[26] S. S. Hafner, S. Nagel, “The electric field gradient at the position of copper in Cu_2_O and electronic charge density analysis by means of K-factors” *Phys. Chem. Miner.* **1983**, *9*, 19–22.

[27] "Cuprous oxide (Cu_2_O) crystal structure, lattice parameters" in *Non-Tetrahedrally Bonded Elements and Binary Compounds I*, vol 41C, Madelung, O., Rössler, U. & Schulz, M. Eds. (Springer-Verlag, Berlin/Heidelberg, 1998) pp. 1-3.

[28] A. Gloystein, N. Nilius, C. Noguera, J. Goniakowski, “Termination-dependent electronic structure and atomic-scale screening behavior of the Cu_2_O(111) surface” *J. Phys. Condens. Matter* **2021**, *33*, 484001.

[29] G. Kresse, J. Hafner, “Ab initio molecular dynamics for liquid metals” *Phys. Rev. B* **1993**, *47*, 558–561.

[30] G. Kresse, J. Furthmüller, “Efficiency of ab-initio total energy calculations for metals and semiconductors using a plane-wave basis set” *Comput. Mater. Sci.* **1996**, *6*, 15–50.

[31] G. Kresse, J. Furthmüller, “Efficient iterative schemes for ab initio total-energy calculations using a plane-wave basis set” *Phys. Rev. B* **1996**, *54*, 11169–11186.

[32] J. P. Perdew, K. Burke, M. Ernzerhof, “Generalized Gradient Approximation Made Simple” *Phys. Rev. Lett.* **1996**, *77*, 3865–3868.

[33] S. Grimme, J. Antony, S. Ehrlich, H. Krieg, “A consistent and accurate *ab initio* parametrization of density functional dispersion correction (DFT-D) for the 94 elements H-Pu” *J. Chem. Phys.* **2010**, *132*, 154104.

[34] S. Grimme, S. Ehrlich, L. Goerigk, “Effect of the damping function in dispersion corrected density functional theory” *J. Comput. Chem.* **2011**, *32*, 1456–1465.

[35] S. L. Dudarev, G. A. Botton, S. Y. Savrasov, C. J. Humphreys, A. P. Sutton, “Electron-energy-loss spectra and the structural stability of nickel oxide: An LSDA+U study” *Phys. Rev. B* **1998**, *57*, 1505–1509.

[36] K. Yu, E. A. Carter, “Communication: Comparing ab initio methods of obtaining effective U parameters for closed-shell materials” *J. Chem. Phys.* **2014**, *140*, 121105.

[37] P. E. Blöchl, “Projector augmented-wave method” *Phys. Rev. B* **1994**, *50*, 17953–17979.

[38] G. Kresse, D. Joubert, “From ultrasoft pseudopotentials to the projector augmented-wave method” *Phys. Rev. B* **1999**, *59*, 1758–1775.

[39] V. Wang, N. Xu, J.-C. Liu, G. Tang, W.-T. Geng, “VASPKIT: A user-friendly interface facilitating high-throughput computing and analysis using VASP code” *Comput. Phys. Commun.* **2021**, *267*, 108033.

[40] Thomas C. Allison, NIST-JANAF Thermochemical Tables - SRD 13, version 1.0.2,

National Institute of Standards and Technology (2013); https://doi.org/10.18434/T42S31

[41] A. A. Peterson, F. Abild-Pedersen, F. Studt, J. Rossmeisl, J. K. Nørskov, “How copper catalyzes the electroreduction of carbon dioxide into hydrocarbon fuels” *Energy Environ. Sci.* **2010**, *3*, 1311–1315.

[42] J. Wellendorff, T. L. Silbaugh, D. Garcia-Pintos, J. K. Nørskov, T. Bligaard, F. Studt, C. T. Campbell, “A benchmark database for adsorption bond energies to transition metal surfaces and comparison to selected DFT functionals” *Surf. Sci.* **2015**, *640*, 36–44.

[43] J. Vickerman, “Model studies on bimetallic Cu/Ru catalysts III. Adsorption of carbon monoxide” *J. Catal.* **1981**, *71*, 175–191.

[44] B. J. Hinch, L. H. Dubois, “First-order corrections in modulated molecular beam desorption experiments” *Chem. Phys. Lett.* **1990**, *171*, 131–135.

[45] D. F. Cox, K. H. Schulz, “Interaction of CO with Cu^+^ cations: CO adsorption on Cu_2_O(100)” *Surf. Sci.* **1991**, *249*, 138–148.

[46] G. Henkelman, B. P. Uberuaga, H. Jónsson, “A climbing image nudged elastic band method for finding saddle points and minimum energy paths” *J. Chem. Phys.* **2000**, *113*, 9901–9904.

[47] T. Brinck, J. H. Stenlid, “The Molecular Surface Property Approach: A Guide to Chemical Interactions in Chemistry, Medicine, and Material Science” *Adv. Theory Simul.* **2019**, *2*, 1800149.

[48] J. H. Stenlid, A. J. Johansson, T. Brinck, “The local electron attachment energy and the electrostatic potential as descriptors of surface–adsorbate interactions” *Phys. Chem. Chem. Phys.* **2019**, *21*, 17001–17009.

[49] J. Halldin Stenlid, F. Abild-Pedersen, “Revealing Local and Directional Aspects of Catalytic Active Sites by the Nuclear and Surface Electrostatic Potential” *J. Phys. Chem. C* **2024**, *128*, 4544–4558.

[50] K. Momma, F. Izumi, “VESTA: a three-dimensional visualization system for electronic and structural analysis” *J. Appl. Crystallogr.* **2008**, *41*, 653–658.

[51] G. Henkelman, A. Arnaldsson, H. Jónsson, “A fast and robust algorithm for Bader decomposition of charge density” *Comput. Mater. Sci.* **2006**, *36*, 354–360.

[52] K. T. Winther, M. J. Hoffmann, J. R. Boes, O. Mamun, M. Bajdich, T. Bligaard, “Catalysis-Hub.Org, an Open Electronic Structure Database for Surface Reactions” *Sci. Data*. **2019**, 6, 75.
